# Attractiveness of black and white modified Shannon traps to phlebotomine sandflies (Diptera, Psychodidae) in the Brazilian Amazon Basin, an area of intense transmission of American cutaneous leishmaniasis

**DOI:** 10.1051/parasite/2017021

**Published:** 2017-06-08

**Authors:** Andreia Fernandes Brilhante, Márcia Moreira de Ávila, Jailson Ferreira de Souza, Antônio Ralph Medeiros-Sousa, Priscila Bassan Sábio, Marcia Bicudo de Paula, Rodrigo Espindola Godoy, Leonardo Augusto Kohara Melchior, Vânia Lúcia Brandão Nunes, Cristiane de Oliveira Cardoso, Eunice Aparecida Bianchi Galati

**Affiliations:** 1 Faculty of Public Health, University of São Paulo São Paulo Brazil; 2 Federal Institute of Acre Rio Branco Acre Brazil; 3 Management of Endemics, City Hall of Xapuri Acre Brazil; 4 Federal University of Acre Rio Branco Acre Brazil; 5 Laboratory of Human Parasitology, Anhanguera-Uniderp University Campo Grande Mato Grosso do Sul Brazil

**Keywords:** Phlebotomine, Attraction, Shannon traps, Anthropophily, American cutaneous leishmaniasis, Amazonia

## Abstract

In the Amazon region the phlebotomine fauna is considered one of the most diverse in the world. The use of Shannon traps may provide information on the anthropophily of the species and improve the traps’ performance in terms of diversity and quantity of insects collected when white and black colored traps are used together. This study sought to verify the attractiveness of the traps to the phlebotomine species of the Brazilian Amazon basin using Shannon traps under these conditions. The insects were collected using two Shannon traps installed side by side, one white and the other black, in a primary forest area of the municipality of Xapuri, Acre, Brazil. Samples were collected once a month during the period August 2013 to July 2015. A sample of females was dissected to test for natural infection by flagellates. A total of 6,309 (864 males and 5,445 females) specimens (36 species) were collected. *Psychodopygus carrerai carrerai* (42%), *Nyssomyia shawi* (36%), and *Psychodopygus davisi* (13%), together represented 90% of the insects collected. *Nyssomyia shawi* and *Psychodopygus davisi* were more attracted by the white color. Specimens of *Nyssomyia shawi*, *Nyssomyia whitmani*, and *Psychodopygus hirsutus hirsutus* were found naturally infected by flagellates in the mid and hindgut. This is the first study in Acre state using and comparing both black and white Shannon traps, demonstrating the richness, diversity, and anthropophilic behavior of the phlebotomine species and identifying proven and putative vectors of the etiological agents of leishmaniasis.

## Abstract (fr)

Dans la région amazonienne, la faune des phlébotomes est considérée comme l’une des plus diversifiées au monde. L’utilisation de pièges Shannon peut fournir des informations sur l’anthropophilie de l’espèce et améliorer les performances des pièges en termes de diversité et de quantité d’insectes recueillis lorsque des pièges de couleur blanche et noire sont utilisés ensemble. Cette étude a cherché à vérifier l’attractivité des pièges pour les phlébotomes du bassin de l’Amazone brésilien en utilisant des pièges Shannon dans ces conditions. Les insectes ont été recueillis en utilisant deux pièges Shannon installés côte à côte, l’un blanc et l’autre noir, dans une zone de forêt primaire de la municipalité de Xapuri, Acre, au Brésil. Des échantillons ont été prélevés une fois par mois pendant la période d’août 2013 à juillet 2015. Un échantillon de femelles a été disséqué pour la recherche d’une infection naturelle par des flagellés. Un total de 6 309 spécimens (864 mâles et 5 445 femelles) de 36 espèces ont été recueillis dans les pièges. *Psychodopygus carrerai carrerai* (42 %), *Nyssomyia shawi* (36 %) et *Psychodopygus davisi* (13 %) représentent ensemble 90 % des insectes récoltés. *Nyssomyia shawi* et *Psychodopygus davisi* étaient plus attirés par la couleur blanche. Des spécimens de *Nyssomyia shawi*, *Nyssomyia whitmani* et *Psychodopygus hirsutus hirsutus* ont été trouvés naturellement infectés par des flagellés dans l’intestin moyen et postérieur. Ceci est la première étude réalisée dans l’état d’Acre à l’aide de pièges Shannon noirs et blancs, qui démontre la richesse, la diversité, le comportement anthropophile des espèces de phlébotomes et qui identifie des vecteurs confirmés et putatifs des agents étiologiques de la leishmaniose.

## Introduction

Phlebotomine flies (Diptera: Psychodidae) are holometabolous insects that are of medical interest mainly because of their involvement in the transmission of etiological agents of leishmaniasis. Currently, in the Neotropics, 520 species have been described and about half of these occur in Brazil [27]. In the Amazon region, the phlebotomine fauna is rich and considered one of the most diverse in the world, with the genera *Psychodopygus*, *Nyssomyia*, and *Trichophoromyia* as the most abundant in entomological surveys [30, 52, 66]. Furthermore, this fauna has a wide variety of incriminated and proven vectors acting in the transmission of leishmaniasis agents, which are commonly represented by different *Leishmania* species, particularly those of the subgenus *Viannia* [42].

The use of different types of traps may provide some information about the diversity of insect species in all kinds of environments. In 1939, Shannon [61] developed a trap using white fabric with light and animal bait to attract mosquitoes. Over the years, and with some modifications, the trap has been used in studies of mosquitoes and phlebotomines with the objective of verifying their anthropophily [46].

The attraction of insects to color was described by Browne and Bennet [8] who found that mosquitoes are more attracted to dark than light colors. However, the species may respond differently to this attraction, as reported by Gilbert and Gouck [32] with regard to mosquitoes and Galati et al. [24, 25], Cruz et al. [17], Moschin et al. [48], and Infran et al. [38] to phlebotomine sandflies.

In Acre (AC) state, little is known about the phlebotomine fly fauna and its behavior. Studies on these insects, undertaken between 2008 and 2016, have only used CDC (Centers for Disease Control) light traps, and have shown a great diversity of species [3, 5, 64, 69]. However, no collections had ever been undertaken in the state with other types of traps, such as the Shannon.

On the basis of this information, and the significant prevalence of cutaneous leishmaniasis in Acre state and in Xapuri municipality (AC), this study sought to verify the phlebotomine species and the traps’ attractiveness to them in the Amazonian forest of the Acre basin, using white and black Shannon traps.

## Methods

### Study area

**Figure 1.**
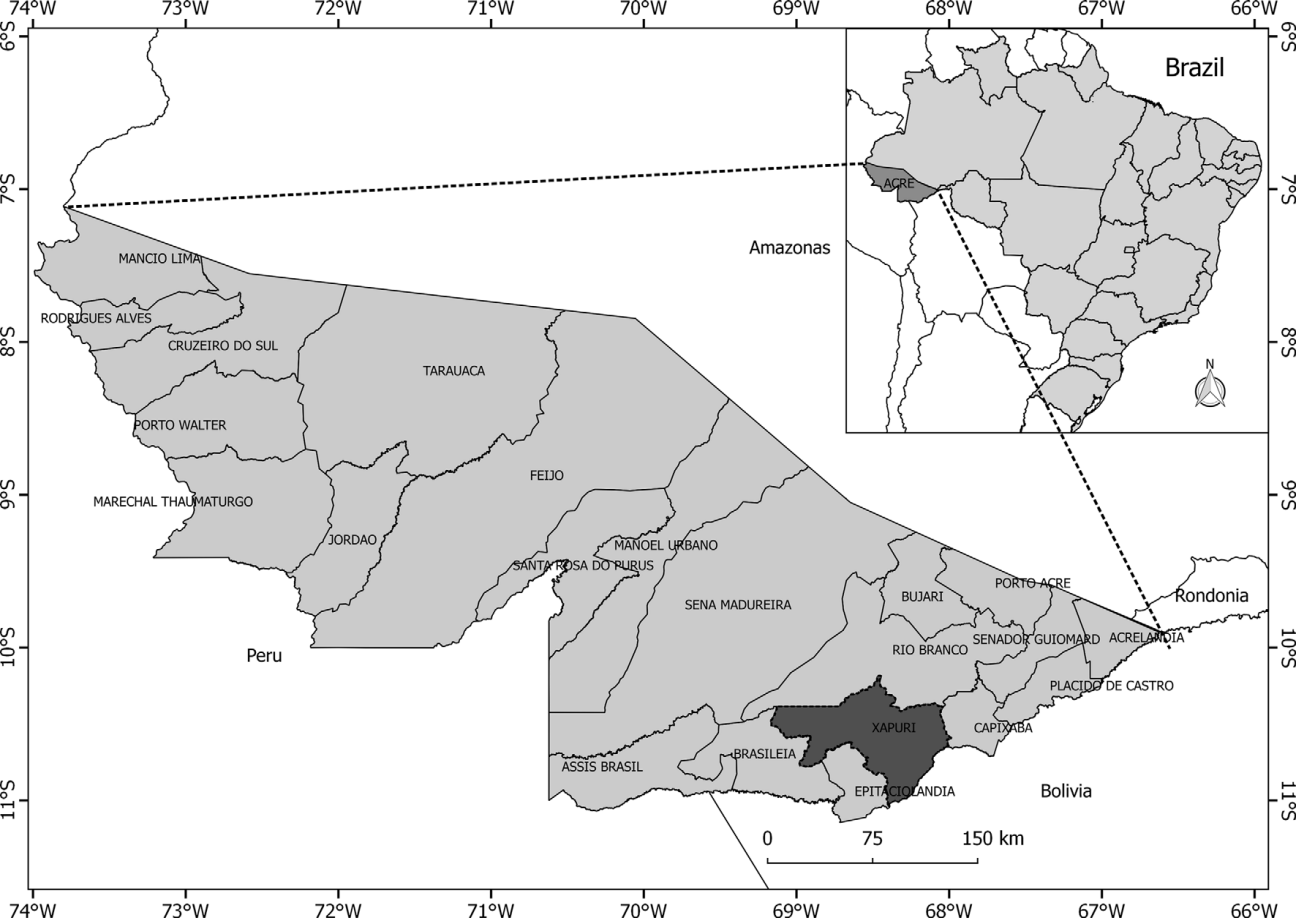
Political map of Brazil, highlighting the Acre state and Xapuri municipality with the study area.

The study was conducted in a rural area about 175 km from Rio Branco, the Acre state capital, in Xapuri municipality, where human and canine cases of American cutaneous leishmaniasis (ACL) have been reported. The municipality is situated in the Vale do Acre mesoregion, and is bordered to the north by the state capital, to the south by Epitaciolândia municipality; to the east by Capixaba and Bolivia; to the west by Brasiléia (Fig. 1) [1, 37]. The primitive vegetation of Xapuri consists of the Amazon biome characterized by a tropical climate with abundant rainfall from October to April and dry months between May and September [19]. The average annual temperature is 27 °C. The human population comprises around 16,000 inhabitants [1, 37].

Xapuri attracts tourists from different parts of the world, motivated by the historical and environmental context which has made the municipality the scene of great conflicts led by the Chico Mendes activist. In addition, it has an ecolodge located in Seringal Cachoeira about 32 km from the urban area of Xapuri, where ecological and environmental tourism activities such as hiking and tree climbing (known as “Arvorismo”) are proposed. This is considered to be the largest circuit in the Amazon, with 600 m in length. The local vegetation consists of native plants and large trees such as *Bertholletia excelsa*, *Hevea brasiliensis*, and *Ceiba pentandra*. *Ceiba pentandra* is popularly known as “Samaúma” and one specimen has become a tourist attraction. More than 500 years old, it is the largest tree in the region at approximately 35 m in height, with a diameter of 25 m [73].

### Phlebotomine collections

**Figure 2.**
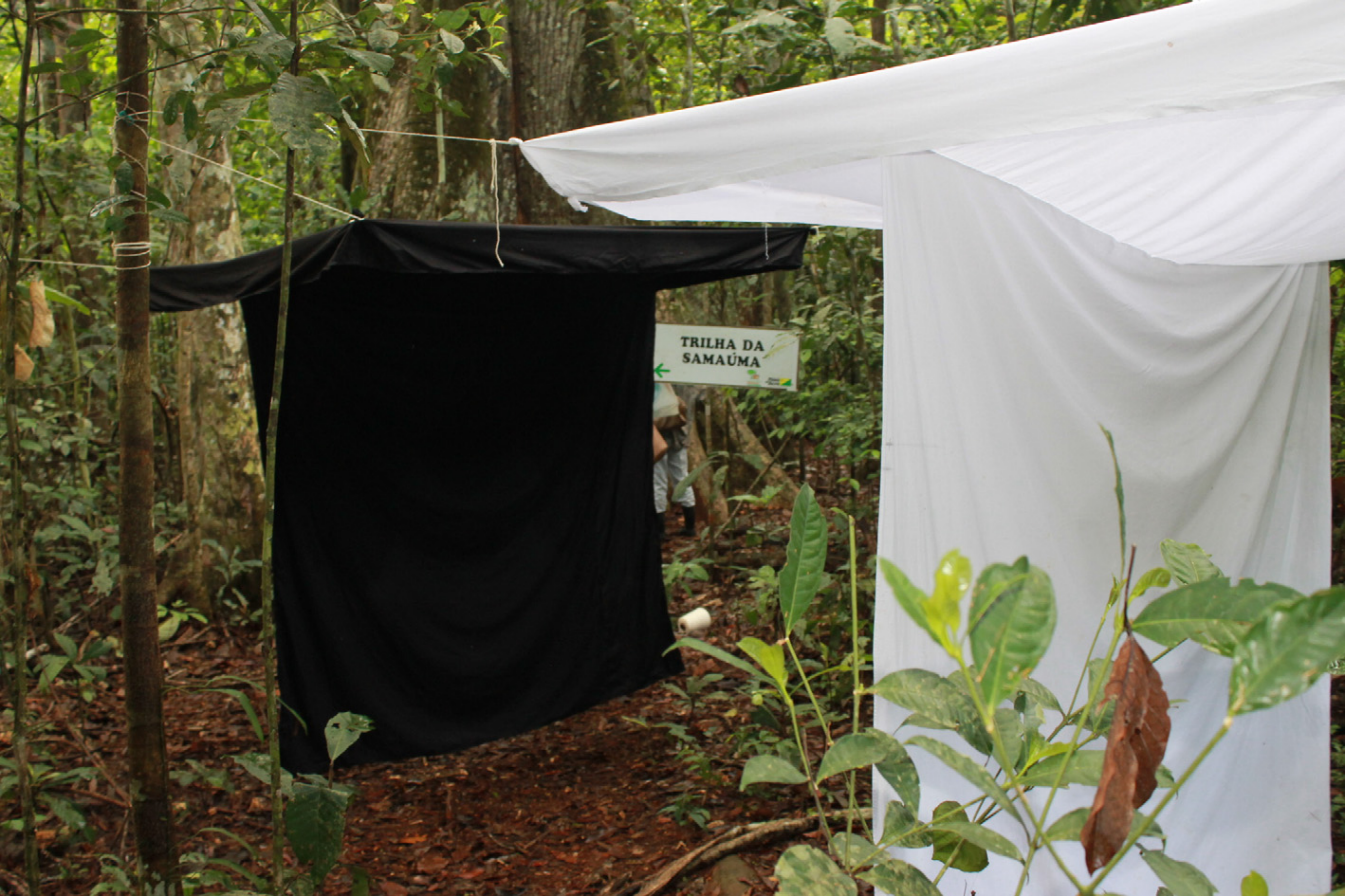
The black and white modified Shannon traps situated in front of the “Samaúma” and near the track area, Xapuri municipality, Acre State, Brazil.

White and black Shannon traps similar to those described by Galati et al. [25] were installed in a primary forest area, side by side at the same distance, about 3 m, from “Samaúma”. The choice of this collection area was due to previous observations of fieldwork, indicating that the Samaúma is a shelter for sandflies (Fig. 2). The sandflies were collected once a month between August 2013 and July 2015, from 6 pm to 10 pm. However, in the months of March 2014, August 2014, April 2015, and July 2015 the collections extended for 24 h uninterrupted. In October 2013, May 2014, and February 2015, due to heavy rains that made it impossible to access the area, the collections were not performed.

Each trap was illuminated at night with a fixed LED (light-emitting diode) light and manual flashlights. The captures were performed using Castro aspirators by two individuals circulating at the same time between the two traps. When the 24-h collections were carried out, the individuals were replaced by other collectors every 6 h. The insects were captured at two-hour intervals and kept in separate vials. They were then taken to the field laboratory, where the vials were opened in a nylon cage (30 cm × 30 cm × 30 cm). Then, female specimens were recaptured and dissected to investigate the presence of flagellates in their guts. The females were immobilized with ethyl acetate and dissected on slides in a drop of sterile saline solution. After their legs and wings were removed, the insects were transferred to another drop of sterile saline. Afterwards, the gut and spermathecae were exposed, covered by a coverslip, and examined under the microscope (400×) to test for natural infection by flagellates and to identify the phlebotomine species. The undissected females and the males were clarified for identification in accordance with Galati’s keys [26] and complementary bibliography [33, 50, 57, 58]. A sample of these clarified insects was mounted in Nelson Cerqueira (NC) resin between slide and coverslip. The remaining insects were examined in eugenol. The abbreviations of the species’ names follow Marcondes [45].

### Data analysis

To confirm sample sufficiency and species richness, the EstimateS program version 9.1.0 was used to plot 1,000 (sample-based and individual-based) randomizations without any replacement and a 95% confidence interval [15].

The hourly rhythm of 24-h collections in dry and rainy months was demonstrated by the proportion rate. The monthly distribution of the species was calculated by Williams’ geometric mean [35].

Shapiro-Wilk normality tests were applied for the abundance data; as none of the variables presented evidence of Gaussian distribution, non-parametric tests were used. A Spearman Rank-order Coefficient (*r*_s_) test was used to analyze the correlation between the three most abundant species and the rainfall. The precipitation data used in this analysis were those of Rio Branco (AC) for a chronological series of the period 1970–2014, obtained by the Civil Defense of the State of Acre. This is because there are no complete data on climatic variables in Xapuri, nor from the nearest meteorological station, Epitaciolândia (AC).

To compare the attractiveness of the traps to the sandflies and the periods of collection (2013–2014 and 2014–2015), a Mann-Whitney test was used for *N* ≥ 10. The significance level in all statistical tests was considered to be *p* < 0.05. The rate of natural infection by flagellates was calculated according to the formula given by Paiva et al. [51].

## Results

A total of 6,309 specimens (864 males and 5,445 females) were attracted to the two traps, constituting a species richness of 36, distributed among four subtribes and 10 genera. The frequencies of the species and female/male ratios collected in the white and black traps are shown in Table 1. *Psychodopygus carrerai carrerai* (41.90%), *Nyssomyia shawi* (36.00%), and *Psychodopygus davisi* (12.52%) predominated, the three together accounting for 90.42% of the insects collected.

**Table 1.** Numbers and percentages of phlebotomines collected with black and white Shannon traps by species, sex and female/male ratio, and species richness (S). Xapuri municipality, Acre state, Brazil, August 2013–July 2015.

Traps	White					Black					Total				
Species	*F*(*d*)	*M*	Total	*F*/*M*	%	*F*(*d*)	*M*	Total	*F*/*M*	%	*F*(*d*)	*M*	Total	*F*/*M*	%
Sex and ratio															
Brumptomyiina															
*Br. pentacantha* (Barretto)	4	–	4	–	0.10	3	1	4	3.00	0.20	7	1	8	7.00	0.12
Lutzomyiina															
*Ev. bacula* (Martins et al.)	–	–	–	–	–	–	1	1	–	0.05	–	1	1	–	0.01
*Ev. tarapacaensis* (Le Pont et al.)	–	–	–	–	–	1	–	1	–	0.05	1	–	1	–	0.01
*Ev. saulensis* (Floch & Abonnenc)	11(1)	–	11	–	0.25	1	–	1	–	0.05	12	–	12	–	0.19
*Ev. termitophila* (Martins et al.),	3(1)	–	3	–	0.07	–	–	–	–	–	3	–	3	–	0.05
*Lu. evangelistai* Martins & Fraiha	1	–	1	–	0.02	2(1)	–	2	–	0.10	3	–	3	–	0.05
*Lu. sherlocki* Martins et al.	5(3)	–	5	–	0.12	5(5)	–	5	–	0.25	10	–	10	–	0.15
Lu. marinkellei* Young	1	2	3	0.50	0.07	1	–	1	–	0.05	2	2	4	1.00	0.06
*Mg. migonei* (França)	1(1)	–	1	–	0.02	–	1	1	–	0.05	1	1	2	1.00	0.03
*Pi. nevesi* (Damasceno & Arouck)	17(2)	2	19	8.50	0.44	7(4)	–	7	–	0.35	24	2	26	12.00	0.41
*Pi. serrana* (Damasceno & Arouck)	1	–	1	–	0.02	2	2	4	1.00	0.20	3	2	5	1.50	0.08
*Pr. choti* (Floch & Abonnenc)	11	2	13	5.50	0.30	3	3	6	1.00	0.30	14	5	19	2.80	0.30
Sergentomyiina															
*Mi. trinidadensis* (Newstead)	–	–	–	–	–	2	–	2	–	0.10	2	–	2	–	0.03
Psychodopygina															
*Ny. antunesi* (Coutinho)	3(2)	2	5	1.50	0.12	3(2)	1	4	3.00	0.20	6	3	9	2.00	0.14
*Ny. fraihai* (Martins et al.)	–	–	–	–	–	7(1)	–	7	–	0.35	7	–	7	–	0.11
*Ny. richardwardi* (Ready & Fraiha)	1	–	1	–	0.02	1	–	1	–	0.05	2	–	2	–	0.03
*Ny. shawi* (Fraiha et al.)	1,517(286)^a^	111	1,628	13.66	37.81	585(165)	53	638	11.03	31.85	2,102	164	2,266	12.81	36.00
*Ny. whitmani* (Antunes & Coutinho)	19(7)^b^	3	22	6.33	0.51	4(3)	4	8	1.00	0.40	23	7	30	3.28	0.45
*Pa. aragaoi* (Costa Lima)	1	–	1	–	0.02	–	–	–	–	–	1	–	1	–	0.01
*Pa. bigeniculata* (Floch & Abonnenc)	–	–	–	–	–	–	1	1	–	0.05	–	1	1	–	0.01
*Pa. dendrophyla* (Mangabeira)	1	1	2	1.00	0.04	–	1	1	-	0.05	1	2	3	0.50	0.05
*Pa. pifanoi* (Ortiz)	–	–	–	–	–	–	1	1	–	0.05	–	1	1	–	0.01
*Ps. amazonensis* (Root)	8	1	9	8.00	0.21	3(3)	–	3	–	0.15	11	1	12	11.00	0.19
*Ps. carrerai carrerai* (Barretto)	1,509(46)	284	1,793	5.31	41.64	723(63)^d^	126	849	5.73	42.38	2,232	410	2,642	5.44	41.90
*Ps. claustrei* (Abonnenc et al.)	3(1)	–	3	–	0.07	–	1	1	–	0.05	3	1	4	3.00	0.06
*Ps. davisi* (Root)	418(16)	78	496	5.35	11.51	240(19)	54	294	4.44	14.67	658	132	790	4.98	12.52
*Ps. hirsutus hirsutus* (Mangabeira)	50(6)	19	69	2.63	1.60	43(8)^c^	8	51	5.37	2.55	93	27	120	3.44	1.90
*Ps. lainsoni* Fraiha & Ward	34(8)	3	37	11.33	0.85	12(10)	2	14	6.00	0.70	46	5	51	9.20	0.81
*Ps. llanosmartinsi* Fraiha & Ward	52(9)	7	59	7.42	1.40	36(12)	7	43	5.14	2.15	88	14	102	6.28	1.61
*Ps. paraensis* (Costa Lima)	2	–	2	–	0.04	–	–	–	–	–	2	–	2	–	0.03
*Ps.* sp. (Guyanensis Series)	2(1)	–	2	–	0.04	2	–	2	–	0.10	4	–	4	–	0.06
*Ps*. sp. (Chagasi series)	1	–	1	–	0.02	–	–	–	–	–	1	–	1	–	0.01
*Th. auraensis* (Mangabeira)	–	29	29	–	0.70	–	15	15	–	0.75	–	44	44	–	0.70
*Th. octavioi* * (Vargas)	–	28	28	–	0.65	–	5	5	–	0.25	–	33	33	–	0.52
*Th. ruifreitasi* Oliveira et al.	–	2	2	–	0.04	–	1	1	–	0.05	–	3	3	–	0.05
*Th. ubiquitalis* (Mangabeira)	4(3)	2	6	2.00	0.14	–	–	–	–	–	4	2	6	2.00	0.09
*Th.* sp	50(8)	–	50	–	1.16	29(11)	–	29	–	1.45	79	–	79	–	1.25
Total	3,730(401)	576	4,306	6.47	100	1,715(307)	288	2,003	6.00	100	5,445	864	6,309	6.30	100
S	31					32					36+				

*Br*. – *Brumptomyia*; *Ev*. – *Evandromyia*; *Lu*. – *Lutzomyia*; *Mg*. – *Migonemyia*; *Mi.* – *Micropygomyia*; *Ny*. – *Nyssomyia*; *Pr.* – *Pressatia*; *Pa*. – *Psathyromyia*; *Ps.* – *Psychodopygus*; *Th.* – *Trichophoromyia*.

* New records for Acre state; M – Male; F – Female; (d) – dissected female;

a, b, c Females infected by flagellates in the mid and hindgut: ^a^(2 females); ^b, c^(1 female);

d A female with the presence of nematode larvae in the head;

+ Females of *Trichophoromyia* sp. probably associated with *Th. auraensis, Th. octavioi,* and *Th. ruifreitasi.*

The species attracted to the black trap were different from those attracted to the white one; three species: *Ps. paraensis*, *Ps. sp*. (Guyanensis series), and *Th. ubiquitalis* were collected exclusively on the white trap and six: *Ev. bacula*, *Ev. tarapacaensis*, *Mi. trinidadensis*, *Ny. fraihai*, *Pa. bigeniculata,* and *Pa. pifanoi* exclusively on the black trap. The black trap attracted one more species than the white (Table 1).

Except for three species of the *Trichophoromyia* genus, *Th. auraensis*, *Th. octavioi,* and *Th. ruifreitasi*, whose females are indistinguishable, among the others attracted, *Evandromyia bacula*, *Psathyromyia bigeniculata*, and *Pa. pifanoi*, the species were represented exclusively by males, while 11 species: *Evandromyia tarapacaensis*, *Ev. saulensis*, *Ev. termitophila*, *Lutzomyia evangelistai*, *Lu. sherlocki*, *Micropygomyia trinidadensis*, *Nyssomyia fraihai*, *Psathyromyia aragaoi*, *Psychodopygus paraensis*, *Psychodopygus* sp. (Chagasi series), and *Psychodopygus sp*. (Guyanensis series) were represented only by females. For the nine most frequent species ≥0.30%, generally the females were more attracted than males to black and white traps. However, for both colors, the difference in attractiveness between sexes was statistically significant (*p* < 0.05) by the Mann-Whitney test only for *Ny. shawi*, *Ps. hirsutus hirsutus*, and *Ps. llanosmartinsi,* and to the white trap for *Ny. whitmani, Ps. davisi*, and *Ps. lainsoni* (Table 2).

**Table 2.** Comparison of the most frequent species of phlebotomines by sex and color of Shannon traps. Values of *p* and *U* by Mann-Whitney test (0.95 level of confidence). Xapuri municipality, Acre state, Brazil, August 2013–July 2015.

Comparison	By sex					
Traps	Black		White		By black and white color	
*U* and *p* values	*U*	*p*	*U*	*p*	*U*	*p*
Species						
*Ny. shawi*	45.0	0.02	59.5	0.00	654	0.16
*Ny. whitmani*	67.0	0.93	21	0.00	198	0.03
*Pi. nevesi*	5.0	0.17	3	0.08	5	0.17
*Pr. choti*	14.5	0.73	8	0.15	45	0.09
*Ps. carrerai carrerai*	127.0	0.05	133	0.07	701.5	0.34
*Ps. davisi*	100.5	0.11	72	0.01	366.5	0.01
*Ps. hirsutus hirsutus*	57.5	0.04	52.5	0.03	321.5	0.22
*Ps. lainsoni*	46.0	0.12	38	0.02	281	0.87
*Ps. llanosmartinsi*	42.5	0.02	36.5	0.01	330.5	0.90

In relation to the color of the traps, the white one was more attractive to all species, but statistically significant by Mann-Whitney test (*p* < 0.05) only for *Ny. whitmani* and *Ps. davisi* (Table 2).

**Figure 3.**
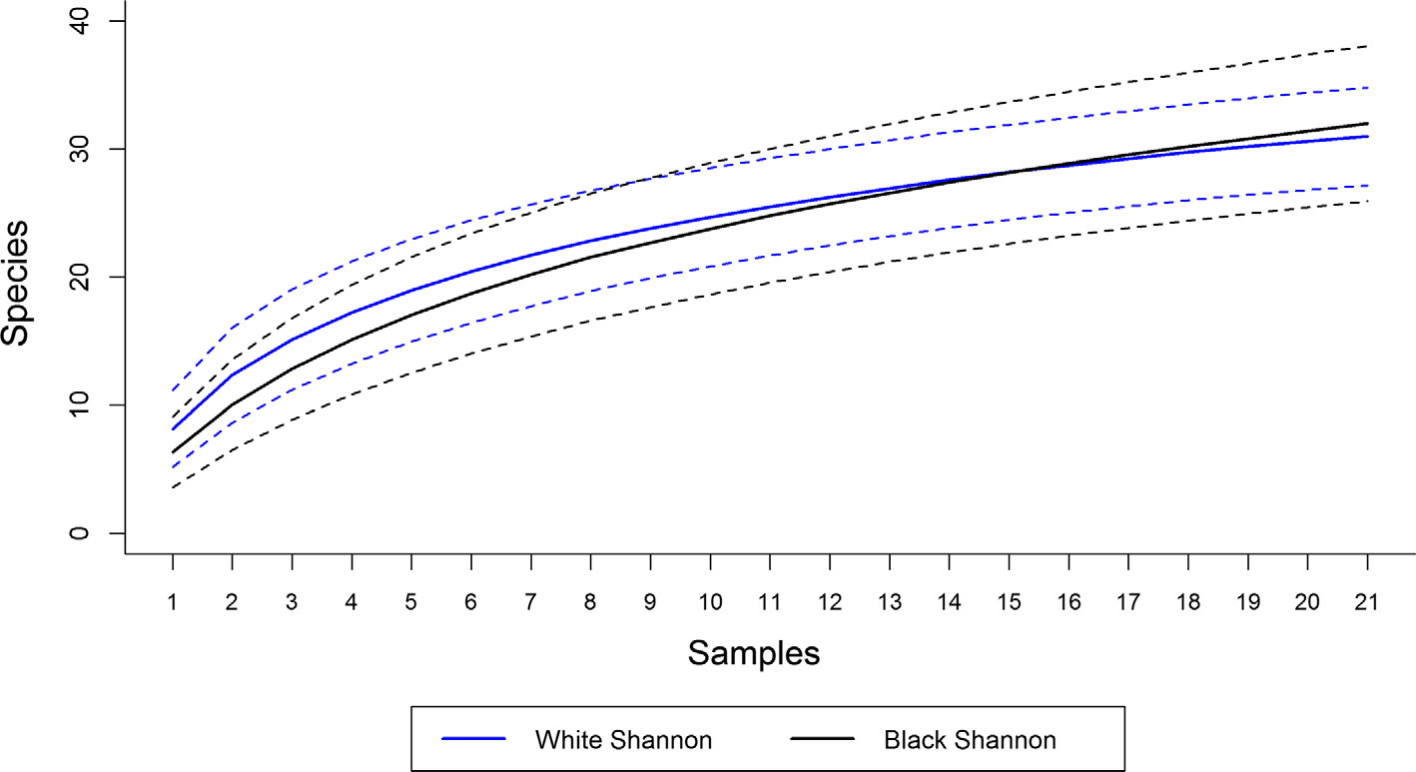
Sample-based species accumulation curves with a 95% confidence interval for white and black Shannon traps, August 2013–July 2015, Xapuri municipality, Acre state, Brazil.

**Figure 4.**
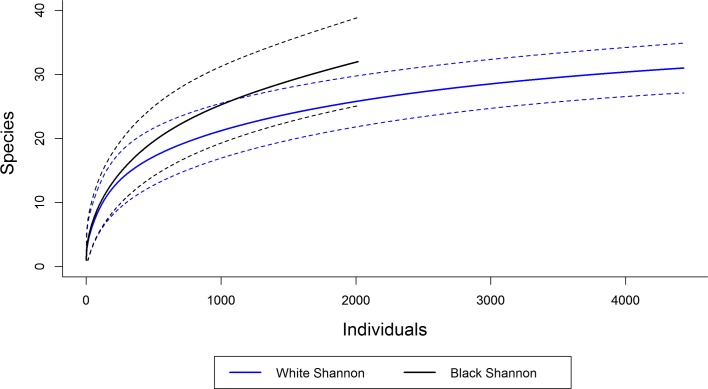
Individual-based species accumulation curves with a 95% confidence interval for white and black Shannon traps, August 2013–July 2015, Xapuri municipality, Acre state, Brazil.

The sample-based species accumulation curves in both traps were close to the asymptote. Both traps showed total overlap in their estimated confidence intervals for number of species, suggesting no differences in observed richness based on samples (Fig. 3). Although the white trap attracted 2.15 times more specimens than the black one (Table 1), the accumulation curve based on individuals shows the black trap having a tendency to attract more species than the white (Fig. 4).

**Figure 5.**
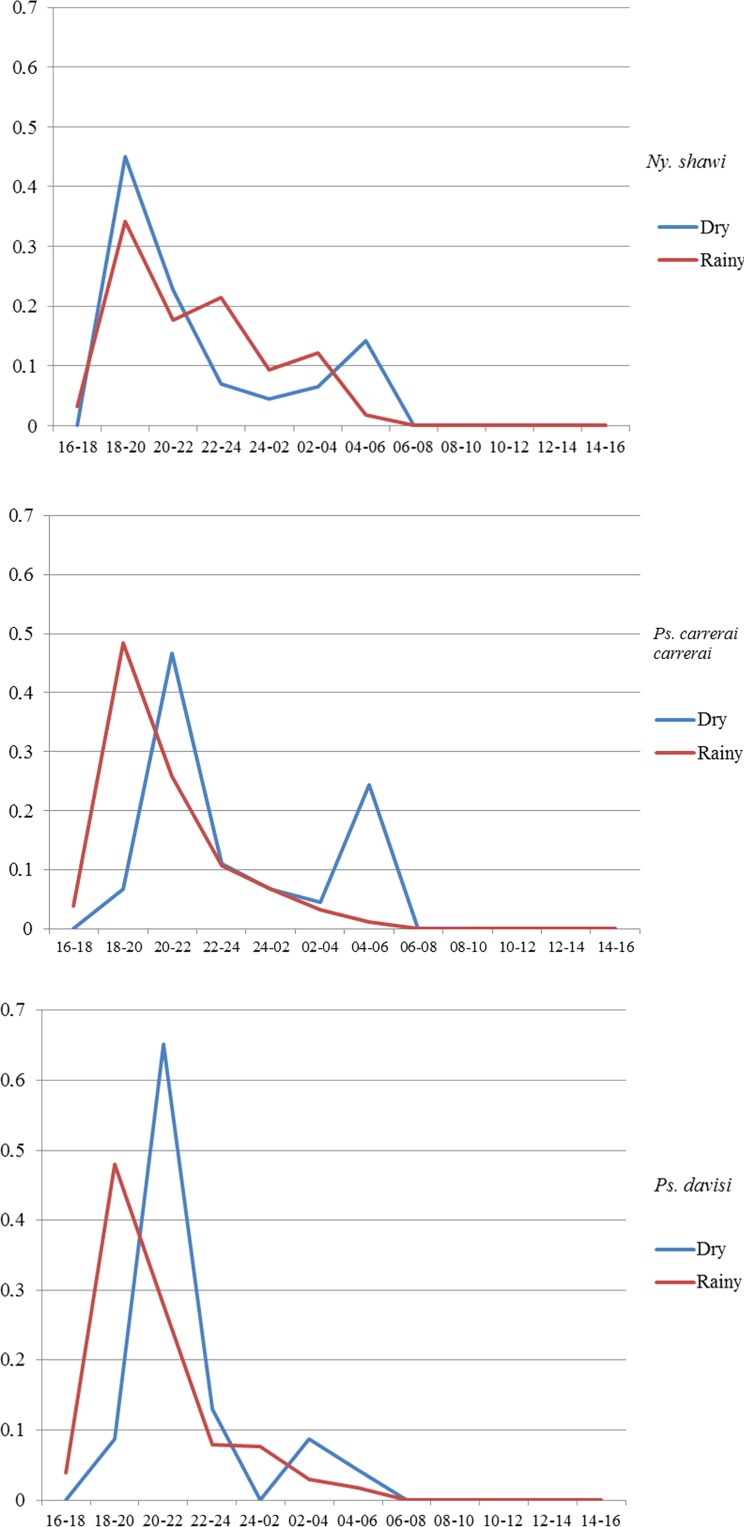
Hourly rhythm of the three most frequent species of phlebotomines by dry and rainy season, in March and August 2014, April and July 2015, in 24-h collections, Xapuri municipality, Acre state, Brazil.

In relation to the hourly rhythm of the most frequent species, *Ny. shawi, Ps. carrerai carrerai*, and *Ps. davisi,* the highest peak occurred in the rainy season between 18 and 20 h, gradually diminishing, however, until 4–6 h. On the other hand, in the dry season, the two *Psychodopygus* species presented their highest peak between 20 and 22 h. Practically no insect was collected during the day time, though in the interval between 18 and 22 h, the three species became active again (Fig. 5).

**Figure 6.**
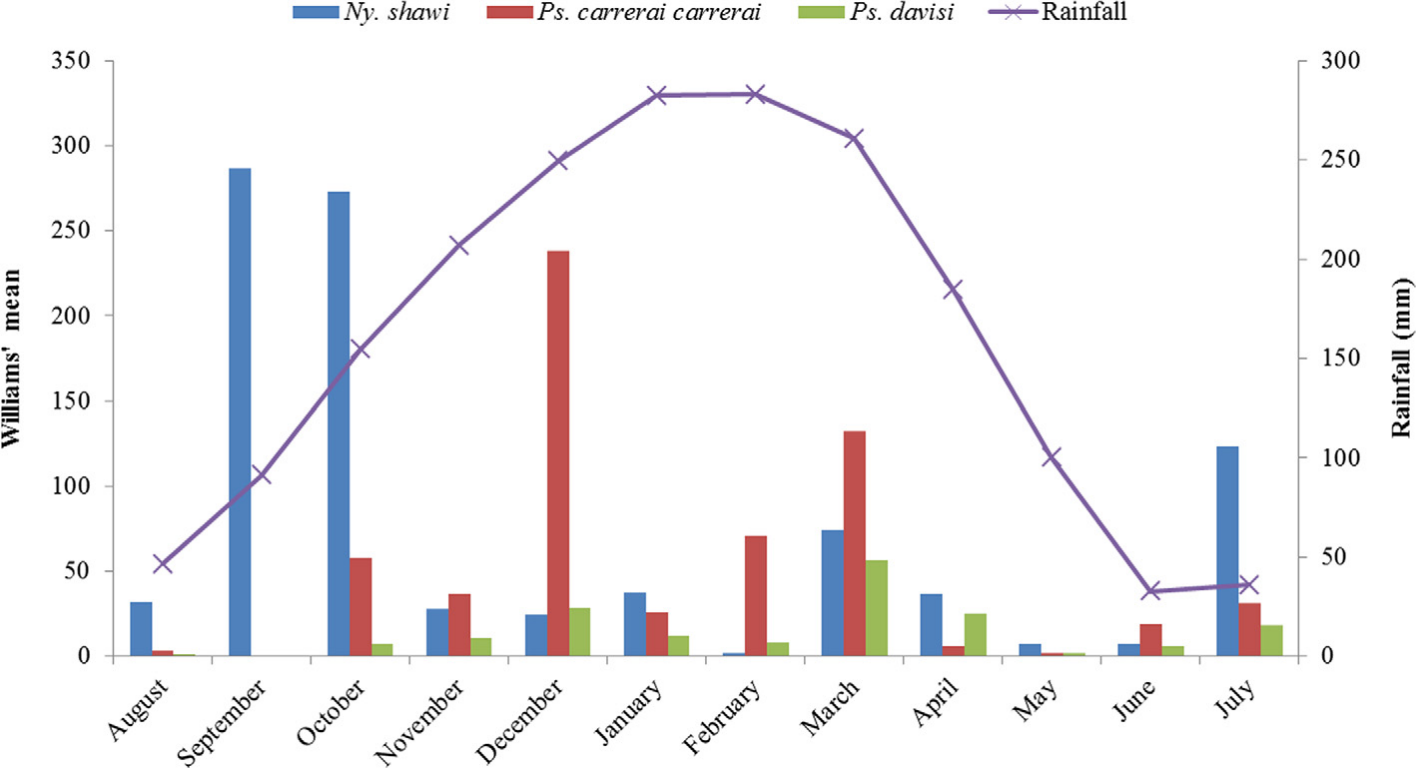
Monthly distribution of Williams’ mean of the three most frequent species in Xapuri municipality and monthly mean rainfall (mm) in Rio Branco municipality, Acre state, Brazil, August 2013–July 2015.

The monthly distribution by Williams’ geometric mean of the species *Ny. shawi*, *Ps. carrerai carrerai*, and *Ps. davisi* is shown in Figure 6. *Nyssomyia shawi* was more frequently collected in the months considered dry, while *Ps. carrerai carrerai* and *Ps. davisi* in the rainy months. No significant correlation was observed between the average number of specimens and rainfall for the species *Ny. shawi* (*r*_s_ = −0.15; *p* = 0.49) and *Ps. davisi* (*r*_s_ = 0.27; *p* = 0.22). However, *Ps. carrerai carrerai* (*r*_s_ = 0.44; *p* < 0.05) presented a significant positive correlation.

Comparing the collection periods August 2013–July 2014 and August 2014–July 2015, it was found that in the former (*n* = 4,239 specimens) twice as many specimens were collected as in the second (*n* = 1,610). However, in the nine months in which there were collections in both periods, no significant difference was observed between the medians of insects attracted in each period (*U* = 25; *p* = 0.19).

A total of 708 females were dissected. *Nyssomyia shawi, Ny*. *whitmani*, and *Ps. hirsutus hirsutus* were found naturally infected by flagellates in the mid and hind portion of the gut. Their flagellate infection rates were 0.44%, 10.00%, and 7.14%, respectively, with a rate of 0.56% in the total dissected females. *Leishmania* DNA was detected by polymerase chain reaction (PCR) in one specimen of *Ps. hirsutus* and another of *Ny. shawi* (Data to be published). A nematode larva was detected in the head of one female of *Ps. carrerai carrerai* and is under investigation by specific molecular diagnosis.

## Discussion

There are few studies on the attraction of phlebotomines by color. However, it is known that in this type of analysis, other factors must be taken into account, such as the environment chosen for the collections, the kind of light source, and the presence of human beings. This is because people also attract these insects, as demonstrated by Perez et al. [53] and Galati et al. [25]. In addition, the use of different traps in entomological surveys may reveal the diversity of species and their characteristic behavior.

The first study using black and white traps in Brazil was undertaken by Galati et al. [25] on the Bodoquena Plateau, midwestern Brazil, where *Lu. almerioi* Galati & Nunes 1999, an anthropophilic species, was significantly more attracted to the black Shannon trap than to the white trap. Recently, studies on the nictemeral rhythm of phlebotomines in a craggy region near the Bodoquena Plateau also found a significant attractiveness of black Shannon traps to *Lu. dispar* Martins & Silva, 1963, another anthropophilic species [38]. These two regions are speleological areas and both species are troglophyles, which may explain their significant attraction to the black Shannon traps.

The attractiveness of black and white Shannon traps to *Ny. neivai* (Pinto 1926) and *Ny. intermedia* (Lutz & Neiva 1912) has also been investigated in agricultural and smallholding areas of the Ribeira Valley, São Paulo state, Brazil. The authors discovered that females of *Ny. intermedia* were more attracted by the black Shannon trap and those of Ny*. neivai* by the white [23, 24]. In another survey conducted in the Serra da Cantareira, metropolitan region of São Paulo, it was found that females of *Pintomyia fischeri* (Pinto 1926) are more attracted to the black Shannon trap, and those of *Migonemyia migonei* to the white. Based on these observations, it was suggested by the authors that *Ny. intermedia* and *Pi. fischeri* may be better adapted to shady and humid locations within forested environments, while *Ny. neivai* and *Mg. migonei* might be more adapted to the anthropic environment [23, 48].

In the central area of Iran, Hesam-Mohammedi et al. [36] demonstrated the efficacy of different methods for sampling phlebotomine sandflies and verified the more numerous presence of *Phlebotomus papatasi* (Scopoli, 1786) and *Phlebotomus sergenti* Parrot, 1917 in black Shannon traps and *Sergentomyia sintoni* Pringle, 1953 in the white. Overall, more specimens were collected in the black trap.

In our study, *Psychodopygus* predominated over other groups in both black (62.8%) and white (57.4%) traps, with the females accounting for 84% of this genus. These findings are consistent with the results of Castellón et al. [13, 14], Cabanillas et al. [11], and Souza et al. [66], who also used white Shannon traps and human bait in Brazilian and Peruvian Amazonia, and found a high density of *Psychodopygus* females. These results differ from those of Alves et al. [2] who collected more males than females, with *Psathyromyia dreisbachi*, *Ps. davisi*, and *Trichopygomyia trichopyga* predominating. Another study with Shannon and CDC traps in the Amazonian biome, and in a transitional area between this system and the Andean biome in Colombia (Putumayo Department), collected 92.5% of the specimens in Shannon traps. Among the specimens collected, only 12% belonged to *Psychodopygus*, though the females also predominated at 86% [7]. In this same study, *Nyssomyia* accounted for 80% of the specimens, with the females representing 84%. These findings differ from our results obtained in Xapuri, where *Nyssomyia* was the second most predominant group, accounting for 36.6%, with the females contributing 92.5% of the specimens.

The phlebotomine fauna of the Acre state is rich and diverse, with 89 species so far recorded [27]. This number is being increased by two: *Lu. marinkellei* and *Th. octavioi* in this report. There are few studies on phlebotomine fauna concerning richness analysis. Results show that high rates of richness and diversity are generally linked to more preserved environments with less anthropic action, as observed by Carvalho et al. [12] in Rio de Janeiro (RJ) and Saraiva et al. [59] in Minas Gerais (MG). Moreover, Feitosa et al. [20], comparing rural and urban environments in Santarém, Pará state, demonstrated by confidence intervals of individual-based rarefaction curves for each area, that urban sites presented the lowest expected number of species. Those of the rural sites attain higher expected values, with fewer individuals. Similar tendencies were observed for white and black traps in our study, which suggests that the black color is more attractive to species with a higher degree of dependence on preserved microenvironments than the white color.

For Xapuri, the curve of species accumulation did not stabilize, which indicates that new species will appear as the collections are made. More collections by Shannon traps are required, as well as with other trap modalities to verify the diversity and richness of species of the locality.

Comparing the numbers of specimens attracted to the traps between the two periods August to July 2013–2014 and August to July 2014–2015, it was observed that a greater number of specimens were collected in the former period. Although the Mann-Whitney test did not show any significant difference between them, based on the results of nine months with collections in both periods, it must be emphasized that the rainy season of 2014–2015 was much more intense than that of 2013–2014, with a historic flood in the state of Acre, including Xapuri where the Acre River borders the municipality. The flood reached a height of 17.62 m while the overflow level is 13.40 m [29], suggesting that this large volume of rainfall may have contributed to the decrease in the phlebotomine population.

The species of the *Nyssomyia* genus collected have already been reported in Acre, Brazil and neighboring countries [5, 10, 11, 27, 63, 70, 71]. *Nyssomyia whitmani* is considered a species complex that is widespread in Brazil and is of epidemiological importance as the main vector of *Leishmania (V.) braziliensis* in this country, and for having been incriminated in the transmission cycle of *L. (V.) guyanensis* and *L. (V.) shawi* in the Amazon region [16, 22, 41, 42, 55]. In this study, this species was more attracted to the white Shannon trap, exactly as has been found in an area in the north of Paraná state [17]. This was different from the results obtained by Galati et al. [25] in areas close to caves in Mato Grosso do Sul state, where it was more frequent in the black trap.

*Nyssomyia shawi* has wide geographical distribution in the Brazilian Amazon, as well as in Bolivia, a country bordering the study region. In Bolivia, it is considered to be a vector of the agents of leishmaniasis due to its great abundance and has been found naturally infected by *Leishmania* (*V*.) *braziliensis* and *L*. (*V*.) *guyanensis* [10, 28, 31, 49]. The captures of this phlebotomine, with high frequencies in the present study, showed no statistically significant difference in the attractiveness to the two traps tested, and also presented a high density of this insect in collections with white traps [26]. Thus, bearing in mind that Shannon traps tend to capture anthropophilic species, the occurrence of high frequencies of *Ny. shawi* in our study, and the finding of specimens with natural infection by flagellates, reinforces these authors’ points of view.

The *Psychodopygus* genus is rich (40 species described so far) and is widely distributed in the Amazon [27, 42]. In this research, *Ps. carrerai carrerai* and *Ps. davisi*, both considered vectors of ACL agents in Brazil, were the dominant species. Highlighting the attractiveness of the white trap to *Ps. davisi,* this species’ anthropophilic behavior and high density in white Shannon traps have been observed in other locations [30, 31, 66, 67]. *Psychodopygus carrerai carrerai* has been collected in both traps and is associated with primary forest environments. It has been reported naturally infected with *Leishmania (V.) braziliensis*, with massive infections in the digestive tract, in research carried out in Bolivia [43, 44]. More often, in northern Brazil, the species *Ps. davisi* has been found infected with *L. (V.) braziliensis*, *L. (V.) guyanensis*, *L. (V.) naiffi*, and *L. (V.) lainsoni* [29, 61, 64, 65, 67], and is considered a potential vector in Rondônia [31] and Pará states [67]. These findings therefore provide strong evidence that these species may also be the vectors in the study area.

In this study, *Ps. hirsutus hirsutus* was found naturally infected by flagellates. Natural infections by trypanosomatids in this species have also been reported by Rangel et al. [56] in Minas Gerais and by Gil et al. [31] in Rondônia, and these infections were attributed by them to *L.* (*V.*) *naiffi*. Recently, this sandfly was found to be infected in Pará state. The isolated parasites were identified by monoclonal antibody testing as *L.* (*V.*) *naiffi* by Souza et al. [66], who suggested that this phlebotomine may be a vector of *L.* (*V.*) *naiffi* in forested areas.

The other species of *Psychodopygus* were less abundant. However, they may have some epidemiological significance because they have been implicated as vectors of *Leishmania* in various locations in northern Brazil and border areas [2, 28, 30, 34, 70].

Regarding the species of the genus *Trichophoromyia*, they are abundant in northern Brazil and neighboring countries. In Acre state and a neighboring country, Peru, *Th. auraensis* has been found in high densities in forest and peri/intradomicilar environments [3, 5, 64], and has been reported as naturally infected by *L.* (*V.) braziliensis*, *L.* (*V.*) *guyanensis*, and *Leishmania* spp. [69, 70]. These observations may suggest that *Th. auraensis* has an important role in *Leishmania* transmission. Concerning *Th. octavioi* and the recently described *Th. ruifreitasi* [50], little is known about their behavior or about their females, herein identified as *Trichophoromyia* sp., because they are morphologically indistinguishable. However, in Xapuri municipality, in our previous studies using CDC traps, there has been a predominance of species of the genus *Trichophoromyia* (based on a sample of 11,295 specimens), while in this study the number of specimens attracted by Shannon traps was low.

The other species only collected in this study in the two traps in small numbers have been reported in other studies using CDC light traps in Acre state [3, 5, 69]. However, we highlight *Pa. pifanoi* which has recently been resurrected from the synonymy of *Pa. shannoni* by Sábio et al. [58] who considered *Pa. cuzquena* to be the former’s junior synonym. The female of this species has been described from specimens collected in the same locality as this study, but using CDC light traps.

According to Moschin et al. [48], the hourly rate may determine the time when the vector-host interaction occurs. In this study, the sandflies’ activity peaks occurred between 18 and 20 h and 04 and 06 h, the same times during which rubber and Brazil-nut extractors work and tourists hike in the area, exposing them to contact with the phlebotomine vectors. In other ACL endemic areas, the nocturnal activity of species of the genera *Psychodopygus* and *Nyssomyia* was observed, with peaks at 18–20 h, 22–00 h, and 04–06 h [34]. In Bolivian forests, the peak of *Ny. shawi* activity has been recorded from 19 to 20 h, a result similar to our findings. It is noteworthy that in this study no phlebotomine activity was observed during the day. However, in some northern regions of Brazil this habit has been registered with regard to some species of the genus *Psychodopygus*, such as *Ps. wellcomei*, *Ps. complexus*, and *Ps. llanosmartinsi* [34, 72].

Concerning the rainfall variable, it seems to influence the populations of *Ps. carrerai carrerai* significantly. In the rainy months, *Ps. carrerai carrerai* and *Ps. davisi* tend to appear in the first few hours of collection, as from 18 h. While *Ny. shawi* appears equally in both periods, but with predominance in the dry months. In other Brazilian Amazonian studies, rainfall and humidity contribute significantly to populations of phlebotomines of the genus *Psychodopygus* [34, 72]. In this study, although rainfall had no significant correlation with the frequency of *Ny. shawi*, there was a high population density of this species in the dry months. This result is similar to observations for other species of the genus *Nyssomyia* in other Brazilian localities [9, 34].

In the Amazon region, the rate of infection by flagellates in phlebotomine flies is high, the most common genera being *Psychodopygus* and *Nyssomyia*, with a great variety of vector species of *Leishmania*. This same scenario was repeated in this study, including the finding of females naturally infected by flagellates of *Leishmania* sp., corroborating the results obtained in other areas of the Brazilian Amazon basin [4, 63, 66].

Here we report the finding of a female specimen of *Ps. carrerai carrerai* with the presence of a nematode, some of which live a part of their life cycle in sandflies [6]. In the Old World, some infestations have been reported in sandflies, some examples of which are *Madathamugadia wanjii* in *Phlebotomus duboscqi* [6], *Didilia* sp., *Didilia ooglypta*, *Psychodiella mackiei* and Tylenchoidea super family in *Phlebotomus papatasi* and *Phlebotomus sergenti* [18, 40, 47, 62, 68], Steinernematidae family in *Phlebotomus tobbi* [39]. In the Americas, experimental infestations by *Anandarema phlebotophaga* and other nematodes have been observed in Colombia and Brazil [54, 60]. In Argentina, wild populations of *Pi. fischeri* have been found parasitized by Tylenchid nematodes [21].

Keeping in mind that Shannon traps tend to attract more anthropophilic species than other types of traps, such as CDC light traps, and that some species were attracted only to the black trap and others only to the white trap, we suggest the combined use of traps of the two colors for a more complete evaluation of the richness, diversity, and anthropophily of phlebotomine fauna.

## Conflict of interest

The authors have no potential conflict of interest.

## References

[1] Acre. Governo do Estado do Acre. [cited 2016 March 21]. Available from: http://www.ac.gov.br/wps/portal/acre/Acre/estado-acre/municipios.

[2] Alves VR, Freitas RA, Santos FL, Oliveira AFJ, Barrett TV, Shimabukuro PHF. 2010 Sand flies (Diptera, Psychodidae, Phlebotominae) from Central Amazonia and four new records for the Amazonas state, Brazil. Revista Brasileira de Entomologia, 56, 220–227.

[3] Araujo-Pereira T, Fuzari AA, Andrade Filho JD, Pita-Pereira D, Britto C, Brazil RP. 2014 Sand fly fauna (Diptera: Psychodidae: Phlebotominae) in an area of leishmaniasis transmission in the municipality of Rio Branco, state of Acre, Brazil. Parasites & Vectors, 7, 2–5. 25103985 10.1186/1756-3305-7-360 PMC4141082

[4] Arias JR, Freitas RA. 1982 The known geographical distribution of sand flies in the state of Acre, Brazil (Diptera: Psychodidae). Acta Amazônica, 12, 401–408.

[5] Azevedo ACR, Costa SM, Pinto MCG, Souza JL, Cruz HC, Vidal J, Rangel EF. 2008 Studies on the sandfly fauna (Diptera: Psychodidae: Phlebotominae) from transmission areas of American cutaneous leishmaniasis in state of Acre, Brazil. Memórias do Instituto Oswaldo Cruz, 103, 760–767. 19148413 10.1590/s0074-02762008000800003

[6] Bain O, Petit G, Paperna I, Finkelman S, Killick-Kendrick M. 1992 A new filarial of lizard transmitted by sandflies. Memórias do Instituto Oswaldo Cruz, 87, 21–29.

[7] Barreto M, Burbano ME, Barreto P. 2000 *Lutzomyia* sand flies (Diptera: Psychodidae) from middle and lower Putumayo Department, Colombia, with new records to the country. Memórias do Instituto Oswaldo Cruz, 95, 633–639. 10998213 10.1590/s0074-02762000000500009

[8] Browne SM, Bennett GF. 1981 Response of mosquitoes (Diptera: Culicidae) to visual stimuli. Journal of Medical Entomology, 18, 505–521. 6121056 10.1093/jmedent/18.6.505

[9] Brilhante AF, Dorval MEC, Galati EAB, Cristaldo G, Rocha HC, Nunes VLB. 2015 Phlebotomine fauna (Diptera: Psychodidae) in an area of fishing tourism in Central-Western Brazil. Revista do Instituto de Medicina Tropical, 57, 233–238. 10.1590/S0036-46652015000300009 PMC4544248 26200964

[10] Bustamante M, Díaz M, Espinoza J, Parrado R, Reithinger R, García AL. 2012 Sand fly fauna in Chapare, Bolivia: an endemic focus of *Leishmania* (*Viannia*) *braziliensis*. Journal of Medical Entomology, 49, 1159–1162. 23025199 10.1603/me12013

[11] Cabanillas M, Braga J, Viena M. 2001 Flebotomíneos da floresta de Terra Firme da Amazonia Peruana (Diptera: Psychodidae). Acta Amazônica, 31, 275–284.

[12] Carvalho BM, Maximo M, Costa WA, De Santanta ALF, Da Costa SM, Da Costa Rego TAN, Pita-Pereira D, Rangel EF. 2013 Leishmaniasis transmission in an ecotourism area: potential vectors in Ilha Grande, Rio de Janeiro state, Brazil. Parasites & Vectors, 6, 325. 24499568 10.1186/1756-3305-6-325 PMC3833291

[13] Castellón EG, Arias JR, Freitas RA, Naiff RD. 1994 Os flebotomíneos da região Amazônica, estrada Manaus – Humaitá, estado do Amazonas, Brasil (Diptera: Psychodidae: Phlebotominae). Acta Amazônica, 24, 91–102.

[14] Castellón EG, Fé NF, Buhrnheim PF, Fé FA. 2000 Flebotomíneos (Diptera: Psychodidae) na Amazônia. II. Listagem das espécies coletadas na Bacia Petrolífera no Rio Urucu, Amazonas, Brasil, utilizando diferentes armadilhas e iscas. Revista Brasileira de Zoologia, 17, 455–462.

[15] Colwell RK, Mao CX, Chang J. 2004 Interpolating, extrapolating, and comparing incidence-based species accumulation curves. Ecology, 85, 2717–2727.

[16] Costa SM, Cechinel M, Bandeira V, Zannuncio JC, Lainson R, Rangel EF. 2007 *Lutzomyia* (*Nyssomyia*) *whitmani* s.l. (Antunes & Coutinho 1939) (Diptera: Psychodidae: Phlebotominae): geographical distribution and the epidemiology of American Cutaneous Leishmaniasis in Brazil. Mini-review. Memórias do Instituto Oswaldo Cruz, 102, 149–153. 17426877 10.1590/s0074-02762007005000016

[17] Cruz CFR, Cruz MFR, Galati EAB. 2013 Sandflies (Diptera: Psychodidae) in rural and urban environments in an endemic area of cutaneous leishmaniasis in southern Brazil. Memórias do Instituto Oswaldo Cruz, 108, 303–311. 10.1590/S0074-02762013000300008 PMC4005571 23778669

[18] Dinesh DS, Kumar V, Das P. 2013 Infestation of Nematodes in *Phlebotomus argentipes* Annandale and Brunetti (Diptera: Psycodidae [sic]), Bihar, India. Global Journal of Medical Research, 13, 7–9.

[19] Duarte AF. 2006 Aspectos da climatologia do Acre, Brasil, com base no intervalo de 1971–2000. Revista Brasileira de Meteorologia, 3, 308–317.

[20] Feitosa MAC, Julião GR, Costa MDP, Belém B, Pessoa FAC. 2012 Diversity of sand flies in domiciliary environment of Santarém, state of Pará, Brazil: species composition and abundance patterns in rural and urban areas. Acta Amazônica, 42, 507–514.

[21] Fernández MS, Santini MS, Diaz JI, Villarquide L, Lestani E, Solomón OD, Achinelly M. 2016 Parasitism by tylenchid nematodes in natural populations of *Pintomyia fischeri* (Diptera: Psychodidae: Phlebotominae) in Argentina. SM Tropical Medicine Journal, 1, 1001.

[22] Freitas RA, Naiff RD, Barrett TV. 2002 Species diversity and flagellate infections in the sand fly fauna near Porto Grande, state of Amapá, Brazil (Diptera: Psychodidae. Kinetoplastida: Trypanosomatidae). Memórias do Instituto Oswaldo Cruz, 97, 53–59. 10.1590/s0074-02762002000100008 11992148

[23] Galati EAB, Marassá AM, Fonseca MB, Gonçalves-Andrade RM, Consales CA, Bueno EFM. 2010 Phlebotomines (Diptera, Psychodidae) in the Speleological Province of the Ribeira Valley: 3. Serra district – area of hostels for tourists who visit the Parque Estadual do Alto Ribeira (PETAR), state of São Paulo, Brazil. Revista Brasileira de Entomologia, 54, 665–676.

[24] Galati EAB, Marassá AM, Gonçalves-Andrade RM, Bueno EFM, Paiva BM, Malafronte RS. 2010 *Nyssomyia intermedia* (Lutz & Neiva) and *Nyssomyia neivai* (Pinto) (Diptera, Psychodidae, Phlebotominae) in a sympatric area: seasonal and nocturnal hourly rhythm in black and white modified Shannon traps. Revista Brasileira de Entomologia, 54, 677–686.

[25] Galati EAB, Nunes VLB, Dorval MEC, Cristaldo G, Rocha HC, Gonçalves-Andrade RM, Naufel G. 2001 Attractiveness of black Shannon trap for phlebotomines. Memórias do Instituto Oswaldo Cruz, 96, 641–647. 11500761 10.1590/s0074-02762001000500008

[26] Galati EAB. 2003 Classificação de Phlebotominae, in Flebotomíneos do Brasil. Rangel EF, Lainson R, Editors Ed. Fiocruz: Rio de Janeiro p. 23–51.

[27] Galati EAB. 2016 Phlebotominae (Diptera, Psychodidae): classificação, morfologia, terminologia e identificação de adultos [apostila], vol. 1, USP: São Paulo.

[28] García AL, Tellez LT, Parrado R, Rojas E, Bermudez H, Dujardin JC. 2007 Epidemiological monitoring of American tegumentary leishmaniasis: molecular characterization of a peridomestic transmission cycle in the Amazonian lowlands of Bolivia. Transactions of Royal Society Tropical Medicine and Hygiene, 101, 1208–1213. 10.1016/j.trstmh.2007.09.002 17945322

[29] Gazeta do Acre. Especial: Uma das piores enchentes da história do Acre. Available from: http://agazetadoacre.com/especial-uma-das-piores-enchentes-da-historia-do-acre/ (dated 02-03-2015; retrieved 11-05-2017).

[30] Gil LHS, Araújo MS, Villalobos JM, Camargo LMA, Ozaki LS, Fontes CJF, Ribolla PEM, Katsuragawa TH, Cruz RM, Almeida e Silva A, Silva LHP. 2009 Species structure of sand fly (Diptera: Psychodidae) fauna in the Brazilian western Amazon. Memórias do Instituto Oswaldo Cruz, 104, 955–959. 20027459 10.1590/s0074-02762009000700002

[31] Gil LHS, Basano SA, Souza AA, Silva MGS, Barata I, Ishikawa EA, Camargo LMA, Shaw JJ. 2003 Recent observations on the sand fly (Diptera: Psychodidae) fauna of the state of Rondônia, Western Amazônia, Brazil: the importance of *Psychodopygus davisi* as a vector of zoonotic cutaneous leishmaniasis. Memórias do Instituto Oswaldo, 98, 751–755. 10.1590/s0074-02762003000600007 14595450

[32] Gilbert IH, Gouck HK. 1957 Influence of surface color on mosquito landing rates. Journal of Economic Entomology, 50, 678–680.

[33] Godoy RE, Galati EAB. 2016 Revalidation of *Nyssomyia fraihai* (Martins, Falcão & Silva 1979) (Diptera: Psychodidae). Journal of Medical Entomology, 53, 1303–1311. 27838613 10.1093/jme/tjw108

[34] Godoy RE, Santana ALF, Graser C, Rangel EF, Vilela ML. 2016 Aspects on the ecology of phlebotomine sand flies (Diptera: Psychodidae) from Guaraí, state of Tocantins, Brazil, endemic area for American cutaneous leishmaniasis. Journal of Medical Entomology, 54, 229–235. 28082651 10.1093/jme/tjw148

[35] Haddow AJ. 1960 Studies on the biting habits and medical importance of East African mosquitos in the genus *Aedes*. I – Subgenera *Aedimorphus*, *Banksinella* and *Dunnius*. Bulletin of Entomology Research, 50, 759–779.

[36] Hesam-Mohammadi M, Rassi Y, Abai MR, Akhavan AA, Rafizadeh S, Sanei Dehkordi A, Sharafkhah M. 2014 Efficacy of different sampling methods of sand flies (Diptera: Psychodidae) in endemic focus of cutaneous leishmaniasis in Kashan district, Isfahan province, Iran. Journal of Arthropod-Borne Disease, 8, 156–162. PMC4478427 26114129

[37] IBGE – Instituto Brasileiro de Geografia e Estatística. 2016 Anuário Estatístico do Brasil [Internet], [cited 2016 October 16]. Available from: www.ibge.gov.br.

[38] Infran JOM, Souza DA, Fernandes WS, Casaril AE, Eguchi GU, Oshiro ET, Fernandes FES, Paranhos Filho AC, Oliveira AG. 2017 Nycthemeral rhythm of phlebotominae (Diptera: Psychodidae) in a craggy region, transitioning between the Wetland and the Plateau, Brazil. Journal of Medical Entomology, 54, 114–124. 28082638 10.1093/jme/tjw151

[39] Karakus M, Arserim SK, Töz SÖ, Özbel Y. 2013 Detection of entomopathogen nematode [EPN-Sand Flies (*Phlebotomus tobbi*)] caught in the wild in Aydin, Kusadasi town and its assessment as a biological control agent. Türkiye Parazitoloji Dergisi, 37, 36–39. 10.5152/tpd.2013.09 23619044

[40] Killick-Kendrick R, Killick-Kendrick M, Qala I, Nawi NA, Ashaford RW, Tang Y. 1989 Preliminary observations on a tetradonematid nematode of phlebotomine sandflies of Afghanistan. Annales de Parasitologie Humaine et Comparée, 64, 332–339.

[41] Lainson R, Shaw JJ, Silveira FT, de Souza AAA, Braga RR, Ishikawa EAY. 1994 The dermal leishmaniases of Brazil, with special reference to the eco-epidemiology of the disease in Amazonia. Memórias do Instituto Oswaldo Cruz, 89, 435–443. 7476229 10.1590/s0074-02761994000300027

[42] Lainson R, Shaw JJ. 2005 New World leishmaniasis, in Microbiology and Microbial Infections, Parasitology. Topley & Wilson’s. Cox FEG, Kreir JP, Wakelin D, Editor Arnold: Sydney, Auckland p. 313–349.

[43] Le Pont F, Breniere RS, Mouchet J, Desjeux P. 1988 Leishmaniose en Bolivie. III. *Psychodopygus carrerai carrerai* (Barretto, 1946) nouveau vecteur de *Leishmania braziliensis braziliensis* en milieu sylvatique de région subandine basse. Comptes Rendus de l’Académie des Sciences – Séries III, 307, 279–282.

[44] Le Pont F, Mouchet J, Desjeux P. 1989 Leishmaniasis in Bolivia – VI. Observations on *Lutzomyia nuneztovari anglesi* Le Pont & Desjeux, 1984 the presumed vector of tegumentary leishmaniasis in the Yungas focus. Memórias do Instituto Oswaldo Cruz, 84, 277–278. 2635754 10.1590/s0074-02761989000200021

[45] Marcondes CB. 2007 A proposal of generic and subgeneric abbreviations of phlebotomines sandflies (Diptera: Psychodidae: Phlebotominae) of the world. Entomology News, 118, 351–356.

[46] Maroli M, Feliciangeli MD, Arias J. 1997 Metodos de Captura, Conservacion y Montaje de los Flebotomos (Diptera: Psychodidae). Documento OPS/HCP/ HCT/95/97, Organizacion Panamericana de la Salud, Washington DC, USA p. 72.

[47] Missiroli A. 1932 Sullo sviluppo di una gregarina del *Phlebotomus*. Annali di Igiene, 42, 373–377.

[48] Moschin JC, Ovallos FG, Sel IA, Galati EAB. 2013 Ecological aspects of phlebotomine fauna (Diptera, Psychodidae) of Serra da Cantareira, Greater São Paulo Metropolitan region, state of São Paulo, Brazil. Revista Brasileira de Epidemiologia, 16, 190–201. 23681335

[49] Ogawa GM, Pereira Júnior AM, Resadore F, Ferreira RGM, Medeiros JF, Camargo LMA. 2016 Sandfly fauna (Diptera: Psychodidae) from caves in the state of Rondônia, Brazil. Revista Brasileira de Parasitologia Veterinária, 25(1), 61–68. 27007243 10.1590/S1984-29612016017

[50] Oliveira AFJ, Teles CBG, Medeiros FM, Camargo LMA, Pessoa FAC. 2015 Description of *Trichophoromyia ruifreitasi*, a new phlebotomine species (Diptera, Psychodidae) from Acre state, Brazilian Amazon. ZooKeys, 526, 65–73. 10.3897/zookeys.526.6128 PMC4607845 26487825

[51] Paiva BR, Secundino NFC, Pimenta PFP, Galati EAB, Andrade-Júnior HF, Malafronte RS. 2007 Standardization of conditions for PCR detection of *Leishmania* spp. DNA in sandflies (Diptera, Psychodidae). Cadernos de Saúde Pública, 23, 87–94. 17187107 10.1590/s0102-311x2007000100010

[52] Pereira Júnior AM, Teles CBG, dos Santos APA, Rodrigues MD, Marialva EF, Pessoa FAC, Medeiros JF. 2015 Ecological aspects and molecular detection of *Leishmania* DNA Ross (Kinetoplastida: Trypanosomatidae) in phlebotomine sandflies (Diptera: Psychodidae) in terra firme and várzea environments in the Middle Solimões Region, Amazonas state, Brazil. Parasites & Vectors, 8, 180. 25889808 10.1186/s13071-015-0789-2 PMC4378226

[53] Perez JE, Villaseca A, Llanos-Cuenta A, Campos M, Guerra H. 1988 Técnicas para colectar “titiras” (*Lutzomyia* spp., Diptera: Psychodidae), em ambientes alto andinos peruanos. Revista Peruana de Entomologia, 30, 77–80.

[54] Poinar GO, Ferro C, Morales A, Tesh RB. 1993 *Anandranema phlebotophaga* n. gen., n. sp. (Allantonematidae: Tylenchida), a new nematode parasite of phlebotomine sand flies (Psychodidae: Diptera) with notes on experimental infections of these insects with parasitic rhabditoids. Fundamental and Applied Nematology, 16, 11–16.

[55] Queiroz RG, Vasconcelos IA, Vasconcelos AW, Pessoa FA, Souza RN, David JR. 1994 Cutaneous leishmaniasis in Ceará state in Northeasten Brazil: incrimination of *Lutzomyia whitmani* (Diptera: Psychodidae) as a vector of *Leishmania braziliensis* in Baturité municipality. The American Journal of Tropical Medicine and Hygiene, 50, 693–698. 8024061 10.4269/ajtmh.1994.50.693

[56] Rangel EF, Ryan L, Lainson R, Shaw JJ. 1985 Observations on the sand fly (Diptera: Psychodidae) fauna of Além Paraiba, State of Minas Gerais, Brazil, and the isolation of a parasite of the *Leishmania braziliensis* complex from *Psychodopygus hirsuta hirsuta*. Memórias do Instituto Oswaldo Cruz, 80, 373–374. 3837173 10.1590/s0074-02761985000300017

[57] Sabio PB, Andrade AJ, Galati EAB. 2014 Assessment of the taxonomic status of some species included in the Shannoni complex, with the description of a new species of *Psathyromyia* (Diptera: Psychodidae: Phlebotominae). Journal of Medical Entomology, 51, 331–341. 24724281 10.1603/me13153

[58] Sábio PB, Brilhante AF, Quintana MG, Andrade AJ, Galati EAB. 2016 On the synonyms of *Psathyromyia* (*Psathyromyia*) *shannoni* (Dyar, 1929) and *Pa. bigeniculata* (Floch & Abonnenc, 1941) and the resuscitation of *Pa. pifanoi* (Ortiz, 1972) with the description of its female (Diptera: Psychodidae: Phlebotominae). Journal of Medical Entomology, 53, 1140–1147. 10.1093/jme/tjw094 27358041

[59] Saraiva L, Silva Reis A, Marteleto NRJ, Sampaio PAA, Rêgo FD, Lima ACVMR, Gontijo CM, Andrade-Filho JD. 2015 Survey of Sand Flies (Diptera: Psychodidae) in an environmentally protected area in Brazil. PLoS One, 10, e0134845. 26267484 10.1371/journal.pone.0134845 PMC4534452

[60] Secundino NFC, Araújo MSS, Oliveira GHB, Massara CL, Carvalho OS, Lanfredi RM, Pimenta PFP. 2002 Preliminary description of a new entomoparasitic nematode infecting *Lutzomyia longipalpis* sand fly, the vector of visceral leishmaniasis in the New World. Journal of Invertebrate Pathology, 80, 35–40. 12234540 10.1016/s0022-2011(02)00046-0

[61] Shannon R. 1939 Methods for collecting and feeding mosquitos in jungle yellow fever studies. The American Journal of Tropical Medicine and Hygiene, 19, 131–148.

[62] Shortt HE, Swaminath CS. 1927 *Monocystis mackiei* n. sp. parasitic in *Phlebotomus argentipes*, Ann. and Brun. The Indian Journal of Medical Research, 15, 539–553. 16789343

[63] Silva TRR, Assis MDG, Freire MP, Rego FD, Gontijo CMF, Shimabukuro PHF. 2014 Molecular Detection of *Leishmania* in sand flies (Diptera: Psychodidae: Phlebotominae) collected in the Caititu indigenous reserve of the municipality of Labrea, state of Amazonas, Brazil. Journal of Medical Entomology, 51, 1–7. 26309318 10.1603/ME14025

[64] Silva-Nunes M, Cavasini CE, Silva NS, Galati EAB. 2008 Epidemiologia da leishmaniose tegumentar e descrição das populações de flebotomíneos no município de Acrelândia, Acre, Brasil. Revista Brasileira de Epidemiologia, 11, 241–251.

[65] Silveira FT, Souza AAA, Lainson R, Shaw JJ, Braga RR, Ishikawa EEA. 1991 Cutaneous leishmaniasis in the Amazon Region: natural infection of the sandfly *Lutzomyia ubiquitalis* (Psychodidae: Phlebotominae) by *Leishmania* (*Viannia*) *lainsoni* in Pará state, Brazil. Memórias do Instituto Oswaldo Cruz, 86, 127–130. 10.1590/s0074-02761991000100021 1842393

[66] Souza AAA, Santos TV, Jennings YLL, Ishikawa EAY, Barata IR, Silva MGS, Lima JAN, Shaw J, Lainson R, Silveira FT. 2016 Natural *Leishmania* (*Viannia*) spp. infections in phlebotomine sand flies (Diptera: Psychodidae) from the Brazilian Amazon region reveal new putative transmission cycles of American cutaneous leishmaniasis. Parasite, 23, 22. 27235194 10.1051/parasite/2016022 PMC4884270

[67] Souza AAA, Silveira FT, Lainson R, Barata IR, Silva MGS, Lima JAN, Pinheiro MSB, Silva FMM, Vasconcelos LS, Campos MB, Ishikawa EAY. 2010 Phlebotominae fauna in Serra dos Carajás, Pará state, Brazil, and its possible implications for the transmission of American tegumentar leishmaniasis. Revista Pan-Amazônica de Saúde, 1, 45–51.

[68] Tang Y, Killick-Kendrick R, Hominick WM. 1997 Life cycle of *Didilia ooglypta* (Nematoda: Tetradonematidae), a parasite of phlebotomine sandflies of Afghanistan. Nematologia, 42, 491–503.

[69] Teles CBG, Santos APA, Freitas RA, Oliveira AFJ, Ogawa GM, Rodrigues MS, Pessoa FAC, Medeiros JF, Camargo LMA. 2016 Phlebotomine sandfly (Diptera: Psychodidae) diversity and their *Leishmania* DNA in a hot spot of American cutaneous leishmaniasis human cases along the Brazilian border with Peru and Bolivia. Memórias do Instituto Oswaldo Cruz, 111, 423–432. 10.1590/0074-02760160054 PMC4957494 27304023

[70] Valdívia HO, de los Santos MB, Fernández R, Baldeviano GC, Zorrilla VO, Vera H, Lucas CM, Edgel KA, Lescano AG, Mundal KD, Graf PCF. 2012 Natural *Leishmania* infection of *Lutzomyia* (*Trichophoromyia*) *auraensis* in Madre de Dios, Peru, detected by a fluorescence resonance energy transfer-based real-time polymerase chain reaction. The American Journal of Tropical Medicine and Hygiene, 87, 511–517. 22802444 10.4269/ajtmh.2012.11-0708 PMC3435357

[71] Vilela ML, Pita-Pereira D, Azevedo CG, Godoy RE, Britto C, Rangel EF. 2013 The phlebotomine fauna (Diptera: Psychodidae) of Guaraí, state of Tocantins, with an emphasis on the putative vectors of American cutaneous leishmaniasis in rural settlement and periurban areas. Memórias do Instituto Oswaldo Cruz, 108, 578–585. 23903972 10.1590/0074-0276108052013007 PMC3970607

[72] Ward RD, Shaw JJ, Lainson R, Fraiha H. 1973 Leishmaniasis in Brazil: VIII. Observations on the phlebotomine fauna of an area highly endemic for cutaneous leishmaniasis, in the Serra dos Carajás, Pará State. Transactions of Royal Society and Tropical Medicine and Hygiene, 67, 174–183. 10.1016/0035-9203(73)90142-9 4784054

[73] Xapuri Info. Xapuri Socioambiental. Seringal Cachoeira: Ecoturismo entre as seringueiras. Available from: http://www.xapuri.info/guiaxapuri/seringal-cachoeira-ecoturismo-entreseringueiras/ (dated 05-10-2015).

